# Integrative analyses of biomarkers and pathways for adipose tissue after bariatric surgery

**DOI:** 10.1080/21623945.2020.1795434

**Published:** 2020-07-20

**Authors:** Yingshan Liu, Jing Jin, Yanshan Chen, Chuna Chen, Zhenguo Chen, Lingling Xu

**Affiliations:** Shenzhen Hospital, Southern Medical University, Shenzhen, China; The Third School of Clinical Medicine, Southern Medical University, Guangzhou, China

**Keywords:** Bariatric surgery, obesity, adipose tissue, differentially expressed genes, DEGs, hub genes, enrichment analyses, PPI network, potential therapeutic agents, bioinformatics

## Abstract

We explored potential biomarkers and molecular mechanisms regarding multiple benefits after bariatric surgery. Differentially expressed genes (DEGs) for subcutaneous adipose tissue (AT) after bariatric surgery were identified by analyzing two expression profiles from the GEO. Subsequently, enrichment analysis, GSEA, PPI network, and gene-microRNAs and gene-TFs networks were interrogated to identify hub genes and associated pathways. Co-expressed DEGs included one that was up-regulated and 22 that were down-regulated genes. The enrichment analyses indicated that down-regulated DEGs were significantly involved in inflammatory responses. GSEA provided comprehensive evidence that most genes enriched in pro-inflammation pathways, while gene-sets after surgery enriched in metabolism. We identified nine hub genes in the PPI network, most of which were validated as highly expressed and hypomethylated in obesity by Attie Lab Diabetes and DiseaseMeth databases, respectively. DGIdb was also applied to predict potential therapeutic agents that might reverse abnormally high hub gene expression. Bariatric surgery induces a significant shift from an obese pro-inflammatory state to an anti-inflammatory state, with improvement in adipocyte metabolic function – representing key mechanisms whereby AT function improves after bariatric surgery. Our study deepens a mechanistic understanding of the benefits of bariatric surgery and provides potential biomarkers or treatment targets for further research.

## 1. Introduction

Obesity has emerged as a worldwide epidemic with far-reaching, increasing, and negative impacts on morbidity from obesity and its co-morbidities that include type 2 diabetes (T2DM), cardiovascular disease (CVD), and several cancer types[1], and of course mortality [2]. The global number of obese individuals (age >5 years) increased from 74 to 440 million for females and 37–357 million in males from 1975 to 2016 [3]. Moreover, 1.30 billion adults and 2.13 billion children and adolescents were also found to be overweight [3]. Obesity is a chronic disease that is caused by multiple factors and characterized as an expansion of adipose tissue (AT) which stores surplus energy, and serves as an active endocrine organ regulating energy homoeostasis and inflammation [4]. Adipocyte inflammation contributes to dysfunction in AT, along with disorders of metabolism, for instance, insulin resistance (IR), dyslipidemia, hyperglycaemia, and hypertension, all jointly accelerating the process of the metabolic syndrome (MetS) [5]. Obesity brings considerable health and economic burdens, compelling us in the clinical and public health settings to seek out efficient weight-loss treatment options.

Reducing energy intake from the diet, increasing exercise training/physical activity and cognitive behaviour training are the cornerstones for weight-loss treatment; nevertheless, poor adherence to lifestyle intervention approaches severely hinders therapeutic efficacy [5] while anti-obesity pharmacotherapy currently shows a limited weight loss effect [6]. Mounting studies provide comprehensive evidence that bariatric surgery is more efficient for obesity [7] than non-surgical treatment, especially for those patients with obesity-associated comorbidities that include T2DM [8] and obstructive sleep apnoea (OSA) [9].

These studies indicate that, compared with non-surgical treatment approaches, bariatric surgery leads to a substantial and durable improvement in body weight, waist circumference, and levels of triglycerides and HDL cholesterol, as well as higher rates of remission in T2DM, OSA and hyperlipidaemia, and better maintenance of therapeutic targets of glycaemic control that would not otherwise be achievable with intensive medical therapy alone. This includes greater reductions in the use of anti-diabetics, anti-hypertensives, and lipid-lowering drugs. In this context, bariatric surgery has rapidly emerged as the most effective intervention today, with positive indicators for subjects with class III obesity (i.e., a BMI >40 kg/m^2^), and is recommended for class I obesity (BMI > 30 kg/m^2^) with co-morbidities like T2DM [10,11].

With continuous improvement seen over the past 20 years, bariatric surgery has entered into an era of laparoscopy, and in this context, Roux-En-Y gastric bypass has been commonly considered as the first choice [12]. The effects of bariatric surgery are far beyond its primary aim of evidentiary weight-loss, extending to metabolic improvements, especially in terms of striking glycaemic control, which exceeds weight loss influence [13], remission of co-morbidities like T2DM [14] and diabetes-related vascular complications [15], improvement of life quality [16], and lower CVD and cancer-associated mortality [17].

The studies above hint at some putative mechanisms on how bariatric surgery affects obesity and its comorbidities efficiently, including modification of cytokine and adipokine profiles, in addition to improving insulin resistance, altered gut hormone release, and eating behaviours. Since obesity is a leading health problem, it is essential to explore potential biomarkers and molecular mechanisms that might be associated with the multifactorial benefits of bariatric surgery and thus move towards the development of effective therapy with corresponding positive metabolic effects.

With the rapid progress and widespread application of high-throughput system (HTS) technologies, integrated bioinformatics analysis has emerged as a promising approach to explore the beneficial mechanism that is apparent following bariatric surgery.

In this study, we identified DEGs for subcutaneous AT after bariatric surgery by analysing two mRNA expression profiles that were downloaded from the GEO database. Subsequently, gene ontology (GO), Kyoto Encyclopedia of Genes and Genomes (KEGG), and GSEA were applied to study the molecular mechanisms for any beneficial effects post-bariatric surgery. Subsequently, we constructed the PPI network and identified the hub genes with Cytohubba. Expression of the hub genes and methylation levels were validated by Attie Lab Diabetes and DiseaseMeth databases. Finally, we analysed the target genes for miRNAs and TFs by use of the NetworkAnalyst database and predicted potential drugs or molecular compounds by the DGIdb database, respectively. Our realization of the mechanisms regarding the benefits of bariatric surgery could be further rationally explored as promising biomarkers or treatment targets for obesity.

## 2. Materials and methods

### 2.1. Microarray data

We selected GEO (http://www.ncbi.nlm.nih.gov/geo), which is a publically available database of gene/microarray profiles for our study.

The search strategy (‘bariatric surgery’ [MeSH Terms] OR bariatric surgery [All Fields]) AND (‘Homo sapiens’[Organism] AND ‘Expression profiling by array’[Filter]) was adopted.

Inclusion criteria were as follows: (i) subcutaneous AT after bariatric surgery from subjects with obesity; (ii) subcutaneous adipose tissue before bariatric surgery used as controls. We extracted the gene expression profiles GSE29409 [18] and GSE59034 [19] from the GEO database. The platform for GSE29409 is GPL7020, NuGO array (human) NuGO_Hs1a520180, which includes five samples of subcutaneous AT that were previously obtained from obese subjects, and five further subcutaneous AT samples in short term after bariatric surgery. The platform for GSE59034 was GPL11532 [HuGene-1_1-st] Affymetrix Human Gene 1.1 ST Array [transcript (gene) version], which includes subcutaneous AT samples from obese subjects before (n = 16) and after short-term bariatric surgery (n = 16). Data table header descriptions and series matrix files of GSE29409 and GSE59034 were downloaded.

### 2.2. Identification of differentially expressed genes

After applying the performance of the Perl script to annotate two databases, we standardized the datasets by quantiles. DEGs with the threshold criterion of |log FC| >1 and p < 0.05 in subcutaneous AT samples after bariatric surgery were screened using the limma V3.42.0 (linear models for microarray data) package of the R software program (version 3.5.0) [20]. Then, the heatmaps of DEGs from each dataset were plotted by the Pheatmap V1.0.12 package [21] again in the R analysis platform. Online tool Draw Venn Diagram (http://bioinformatics.psb.ugent.be/webtools/Venn/) was applied to detect common DEGs among the two datasets.

### 2.3. Functional and pathway enrichment analysis

GO analysis is used extensively to identify the characteristic biological attributes of genes, gene products, and sequences, including biological process (BP), cell components (CC) and molecular function (MF) [22]. KEGG analysis provides a comprehensive set of biointerpretation of genomic sequences and protein interaction network information [23].

In this study, GO terms and KEGG pathway enrichment analysis of DEGs were automatically completed and visualized by the clusterProiler V3.14.0 [24], digest V0.6.23, and the Goplot V1.0.2 package [25] in the R software statistical analysis platform (significant as p < 0.05 and a q-value <0.05) and the CluePledia plug-in [26] and Cluego plug-in [27] in Cytoscape software [28] (version 3.7.1, http://www.cytoscape.org/) (with a kappa score ≥0.4).

### 2.4. Gene set enrichment analysis

Further GSEA was carried out for all genes that were detected by use of GSEA software (version 4.0.0) [29], providing us another option to screen out significant differential biological functions derived after bariatric surgery. The gene set arrangement was performed 1000 times per analysis. Gene sets were considered to be significantly enriched with an alpha or P-value <5% and a false discovery rate (FDR) <25%.

### 2.5. Construction of PPI network and identification of hub genes

We searched STRING (http://www.string-db.org/), which is an online tool to identify and predict interactions between genes or proteins, to construct the PPI network of DEGs with the cut-off standard as a combined score >0.4. Next, Cytoscape software was used to visualize the PPI network for DEGs. The MCODE (Molecular Complex Detection) V1.5.1, which is a plug-in of Cytoscape, was applied to identify significant modules (MCODE score ≥4) [30]. Moreover, Cytohubba [31], which is another plug-in of Cytoscape, was employed to study essential nodes in the network with 11 methods (MCC exhibits a satisfied comparative performance) completed to explore the hub genes that were contained in the PPI network.

### 2.6. Expression Levels of Hub Genes in Obesity

We searched the Attie Lab Diabetes database (http://diabetes.wisc.edu), which is an open-source database displaying differential gene expression profiles in six tissues (adipose included) of both lean and obese BTBR mice and different ages (i.e., at 4 and 10 weeks) [32], to verify the mRNA levels of hub genes in obesity (significance set at p < 0.05).

### 2.7. Methylation analysis of hub genes in T2DM

DiseaseMeth 2.0 (http://bioinfo.hrbmu.edu.cn/diseasemeth/) was utilized to annotate the methylation levels of TYROBP, TLR8, FCER1 G, HCK, NCF2, CTSS, FCGR2A, MNDA, and C3AR1 in T2DM. The analysis was performed as previously described and compared with the Student’s t-test when comparing T2DM and the normal group.

### 2.8. Construction of the target gene-miRNA network and the target gene-TF network

MiRNA or TF control gene expression under defined disease conditions through interaction with target-genes during the post-transcriptional stage were analysed [33,34]. We applied NetworkAnalyst (https://www.networkanalyst.ca/) [35] to integrate miRNA databases; we also used miRTarBase (http://mirtarbase.mbc.nctu.edu.tw/php/download.php) and TarBase (http://diana.imis.athena-innovation.gr/DianaTools/index.php?r=tarbase/index), and TF databases ENCODE (http://cistrome.org/BETA/). We visualized target gene-miRNA and gene-TF networks by employing Cytoscape software.

### 2.9. Identification of the potential drugs

The Drug Gene Interaction Database (DGIdb) version 3.0.2 (https://www.dgidb.org) [36] is an available resource of the drug targeted and sensitive genome and drug–gene interaction. We searched the DGIdb to predict potential drugs or molecular compounds that interacted with the DEGs and visualized the drug–gene interaction network by the Cytoscape software.

## 3. Results

### 3.1. Identification of DEGs after bariatric surgery

**Figure 1.**
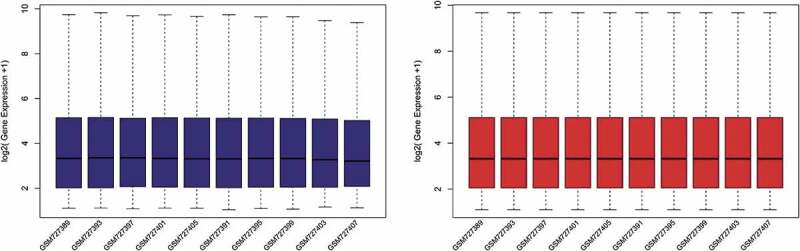
Box plots of the gene expression data before and after normalization. (a) Standardization of GSE29409, (b) standardization of GSE59034. The x-axis label represents the sample symbol and the y-axis label represents the gene expression values. The black line in the box plot represents the median value of gene expression. The blue bar represents the data before normalization, and the red bar represents the data after normalization

**Figure 2.**
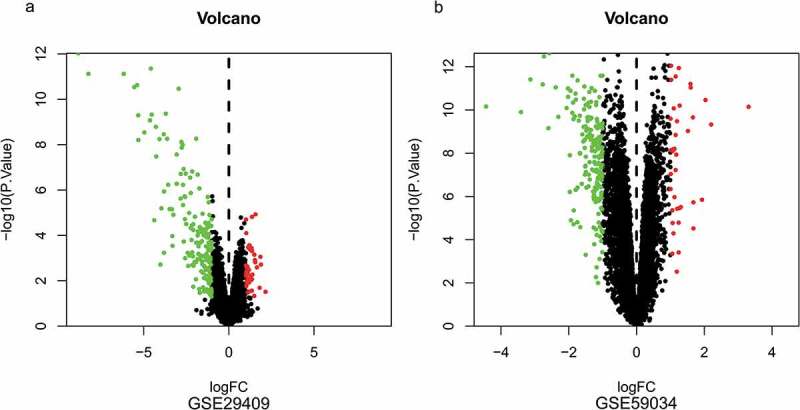
Volcano plots of differentially expressed genes. (a) GSE29409, (b) GSE59034. Data points in red represent up-regulated, and green represent down-regulated genes. The differences are set as |log FC|>1

**Figure 3.**
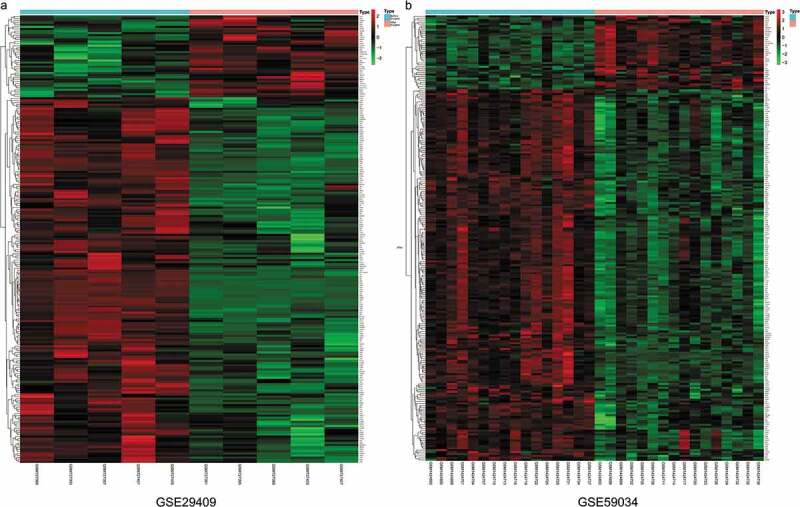
Heatmap of differentially expressed genes identified in (a) GSE29409, (b) GSE59034. Legend on the top right indicates the log fold change of the genes

**Figure 4.**
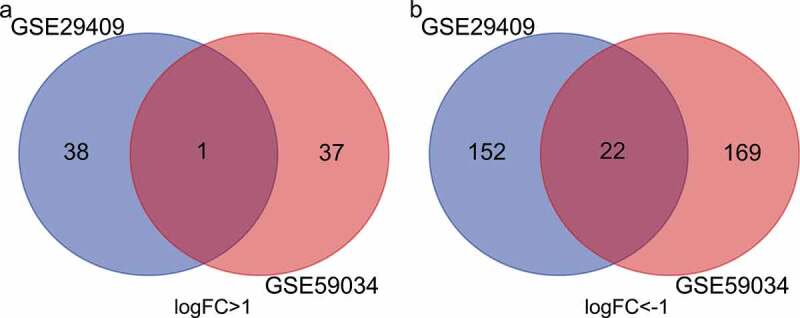
Venn diagram of common differentially expressed genes from the two datasets. (a) 1 DEG was up-regulated in the two datasets, (b) 2 DEGs was down-regulated in the two datasets

To identify DEGs before and after bariatric surgery, and after standardization of the datasets (Figure 1), 213 and 229 DEGs were extracted from GSE29409 and GSE59034 based on the defined criteria, respectively. The DEGs are shown in the volcano plots and the heatmaps whose clustering was performed with Euclidean distance (Figures 2 and 3), of which GSE29409 included 39 up-regulated genes and 174 down-regulated genes, and GSE59034 included 38 up-regulated genes and 191 down-regulated genes are also shown. The co-expressed DEGs were integrated using the Venn Diagram online tool, including one up-regulated and 22 down-regulated genes in subcutaneous AT after bariatric surgery (Figure 4, and Table 1).

**Table 1.** Differentially expressed genes after bariatric surgery

DEGs	Genes name
Up-regulated	FGFBP2
Down-regulated	ALOX5 AMICA1 AQP9 C1orf162 C1QB C3AR1 C5AR1 CECR1 CTSS CXCL16 CYTIP EVI2A FCER1 G FCGR2A GPR183 HCK MNDA NCF2 RGS1 S100A8 TLR8 TYROBP

### 3.2. Gene ontology and KEGG pathway enrichment analyses after bariatric surgery

**Figure 5.**
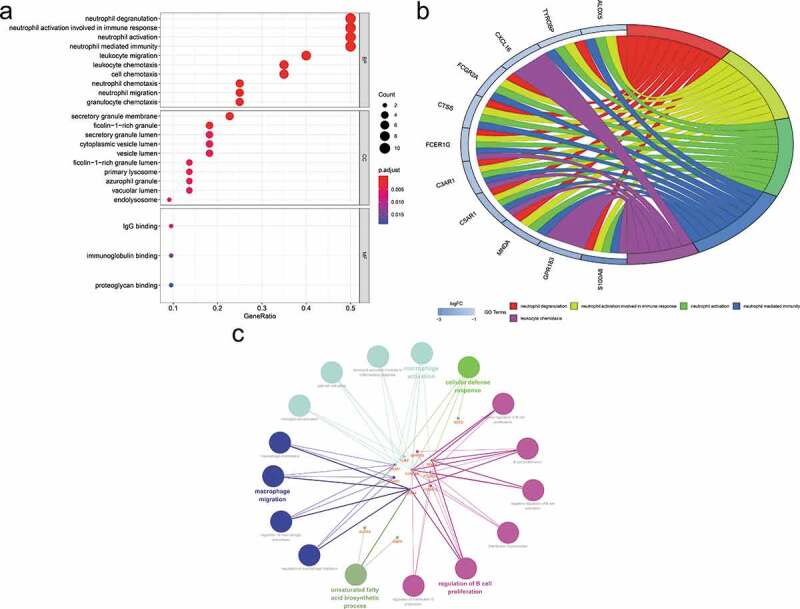
Gene Ontology (GO) enrichment analysis of differentially expressed genes (DEGs). (a) Advanced bubble chart shows GO enrichment significance items of DEGs in three functional groups: molecular function (MF), biological processes (BP), and cell composition (CC). The x-axis label represents the gene ratio, and the y-axis label represents GO terms. (b) Chord plot shows the distribution of DEGs in different GO-enriched functions. Symbols of DEG are presented on the left side of the graph with their fold change values mapped by colour scale. Gene involvement in the GO terms was determined by coloured connecting lines. (c) Cluego network diagram shows the relationship between the DEGs and GO terms

**Figure 6.**
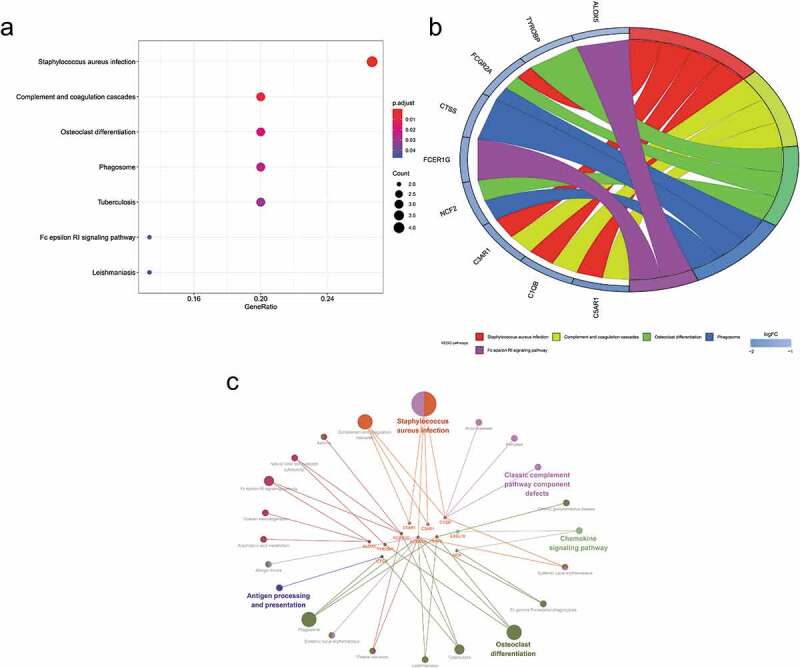
Kyoto Encyclopaedia of Genes and Genomes (KEGG) pathway analysis of differentially expressed genes (DEGs). (a) Advanced bubble chart shows enrichment of DEGs in signalling pathways. The x-axis label represents the gene ratio and the y-axis label represents pathway. (b) Chord plot shows the distribution of DEGs in different KEGG pathways. Symbols of DEG are presented on the left side of the graph with their fold change values mapped by colour scale. Gene involvement in the KEGG pathways was determined by coloured connecting lines. (c) Cluego network diagram shows the relationship between the DEGs and KEGG pathways

The enriched GO and KEGG pathway analysis of 22 down-regulated genes were analysed and visualized in clusterProiler, Digest, and Goplot packages of the R software analysis package and CluePledia plug-in and Cluego plug-in of Cytoscape software (Figures 5, and 6, Table S1 and S2). The result of GO enrichment indicated that for BP, down-regulated DEGs were significantly enriched in neutrophil degranulation, activation, migration and chemotaxis that play key roles in the host immune response, neutrophil-mediated immunity, leukocyte migration and chemotaxis, and general cellular chemotaxis. Regarding CC, DEGs were significantly enriched in the vacuolar lumen, azurophil granule, primary lysosome, ficolin-1-rich granule lumen, vesicle lumen, cytoplasmic vesicle lumen, secretory granule lumen, ficolin-1-rich granule, secretory granule membrane, and endolysosome. For MF, down-regulated genes were significantly enriched in IgG binding, immunoglobulin binding, and proteoglycan binding. The result of KEGG pathway-enrichment indicated that DEGs were mainly enriched in the complement and coagulation cascades, the phagosome, and the Fc epsilon RI signalling pathway.

### 3.3. GSEA analysis

**Figure 7.**
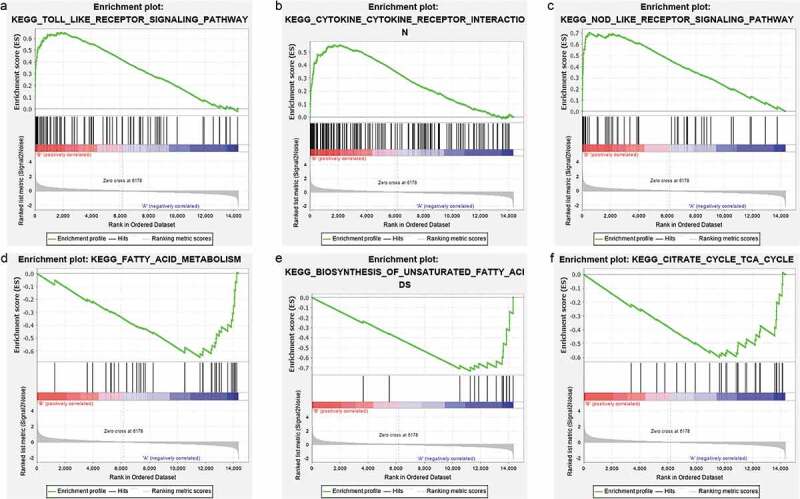
GSEA plots showing the most enriched gene sets of all detected genes in the obese subjects before and after bariatric surgery in the GSE29409 dataset. The top 3 most significant up-regulated enriched gene sets in the before bariatric surgery group: (a) toll-like receptor signalling pathway, (b) NOD-like receptor signalling pathway, (c) cytokine-cytokine receptor interaction. The top 3 most significant up-regulated enriched gene sets in the after bariatric surgery group: (d) fatty acid metabolism, (e) biosynthesis of unsaturated fatty acids, (f) citrate cycle (TCA cycle)

GSEA was carried out to identify the possible mechanism on how bariatric surgery is effective. Samples were divided into a before bariatric surgery group and an after bariatric surgery group. The analysis indicated that the most significant-enriched gene sets positively correlated with the before bariatric surgery group, which included the toll-like receptor signalling pathway, the NOD-like receptor signalling pathway, the cytokine-cytokine receptor interaction effect, the chemokine signalling pathway, autoimmune thyroid disease, the B cell receptor signalling pathway, Fc gamma R-mediated phagocytosis, the Jak-STAT signalling pathway, type 1 diabetes mellitus, natural killer cell-mediated cytotoxicity, and leukocyte transendothelial migration. Moreover, the most enriched gene sets with a significant difference that were positively correlated with the after bariatric surgery group, were fatty acid metabolism, biosynthesis of unsaturated fatty acids, the citrate cycle (TCA cycle), valine, leucine and isoleucine degradation, pyruvate metabolism, aminoacyl-tRNA biosynthesis, and glycolysis/gluconeogenesis (Figure 7).

### 3.4. PPI network analysis and hub gene selection

**Figure 8.**
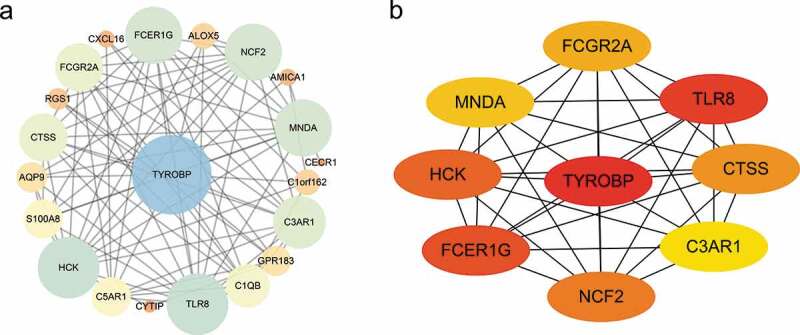
Protein-protein interaction (PPI) network. (a) PPI network of differentially expressed genes (DEGs), (b) subnetwork of top nine hub genes from the PPI network. Node colour reflects the degree of connectivity (Red colour represents a higher degree, and yellow colour represents a lower degree)

As shown in Figure 8(a), the PPI network of DEGs, which was based on STRING included 22 down-regulated genes that were gathered as a cluster consisting of 21 nodes and 85 edges. MCODE was applied to identify the most significant module that was comprised of 13 nodes, which were all down-regulated DEGs (MCODE score = 10). The top nine hub genes selected by the MCC method (score≥5000) and node degree (score ≥10) in the Cytohubba plug-in included TYROBP, TLR8, FCER1 G, HCK, NCF2, CTSS, FCGR2A, MNDA, C3AR1 (Figure 8(b)).

### 3.5. Expression levels of hub genes in obesity

**Figure 9.**
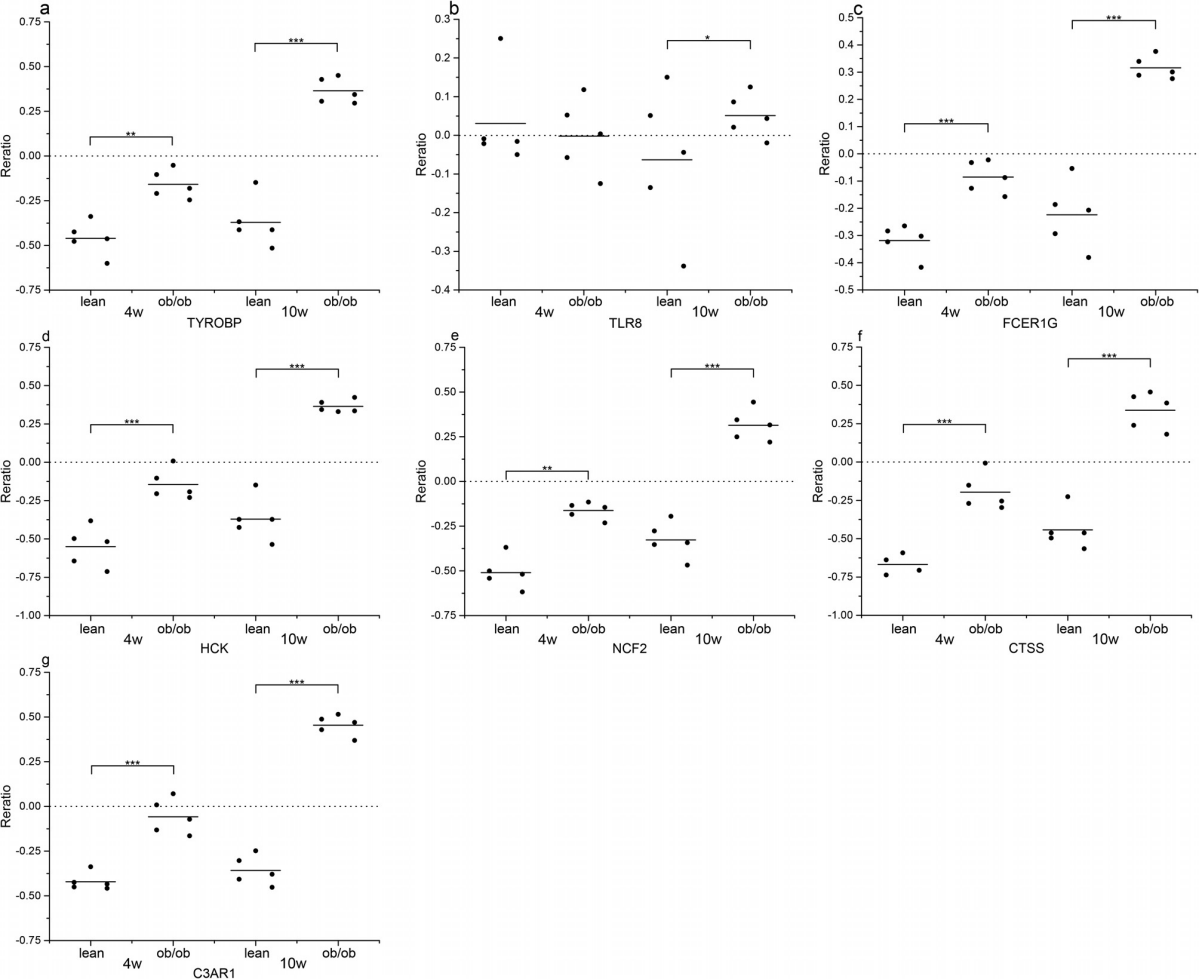
The expression of genes (a) TYROBP, (b) TLR8, (c) FCER1 G, (d) HCK, (e) NCF2, (f) CTSS, and (g) C3AR1, significantly up-regulated in the adipose of the 10-weeks obese diabetic mice. *** p < 0.001, ** p < 0.01, *p < 0.05

We applied the Attie Lab Diabetes database to verify the hub gene mRNA levels in obesity, which indicated that expression of TYROBP, TLR8, FCER1 G, HCK, NCF2, CTSS and C3AR1 was significantly up-regulated in 10-week obese mice as compared to the lean group (Figure 9).

### 3.6. Methylation analysis of hub genes in T2DM

**Figure 10.**
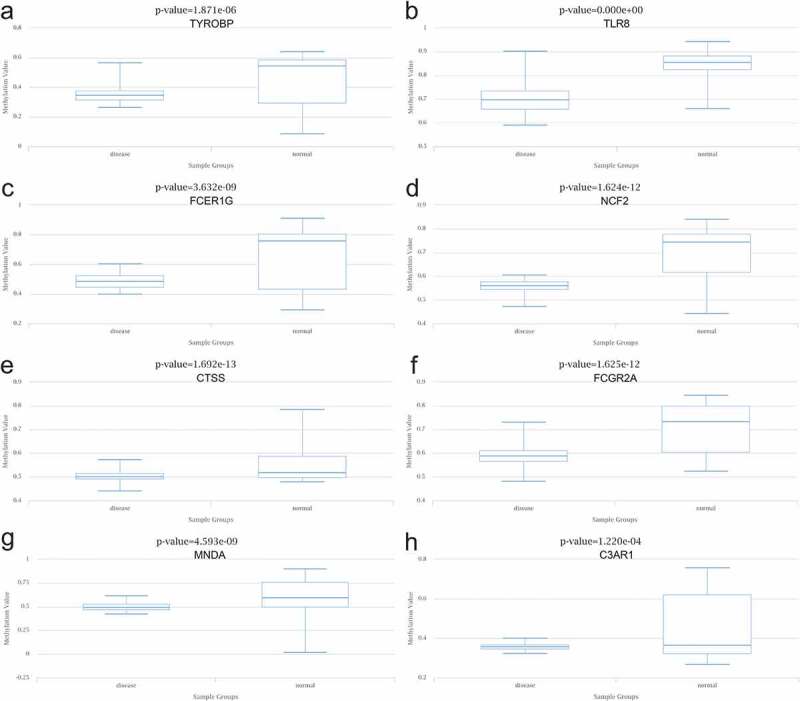
Methylation analysis of 8 hub genes (a) TYROBP, (b) TLR8, (c) FCER1 G, (d) NCF2, (e) CTSS, (f) FCGR2A, (g) MNDA, and (h) C3AR1 in type 2 diabetes (left) vs normal (right)

The data aligned to the methylation levels of nine hub genes in T2DM were obtained from DiseaseMeth 2.0, indicating significant hypomethylation levels of TYROBP, TLR8, FCER1 G, NCF2, CTSS, FCGR2A, MNDA, and C3AR1 in T2DM. Inversely, the methylation level of HCK was significantly higher in the setting of T2DM (Figure 10).

### 3.7. Construction of the target gene-miRNA network and the target gene-TF network

**Figure 11.**
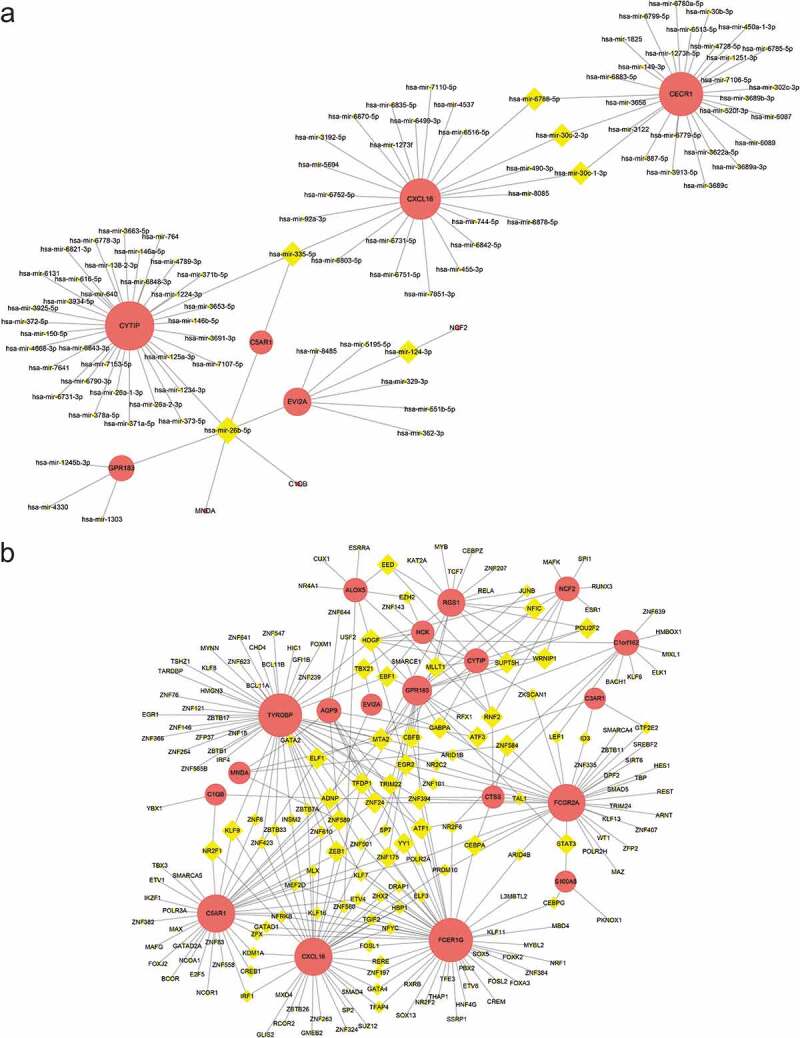
The networks of (a) target gene-miRNA and (b) target gen-TF. The red circle nodes are the genes, and yellow diamond nodes are the miRNAs

The top three targeted DEGs for miRNAs were CYTIP that was modulated by 36 miRNAs, CECR1 that was modulated by 29 miRNAs, and CXCL16 that was modulated by 25 miRNAs. The miRNA that may control the largest number of DEGs (six genes) was hsa-mir-26b-5p (Figure 11(a)). The top five targeted DEGs for TFs were FCER1 G that was modulated by 51 TFs, TYROBP that was modulated by 50 TFs, C5AR1 that was modulated by 38 TFs, that was CXCL16 modulated by 38 TFs, and FCGR2A that was modulated by 37 TFs (Figure 11(b)).

### 3.8. Identification of the potential drugs

**Figure 12.**
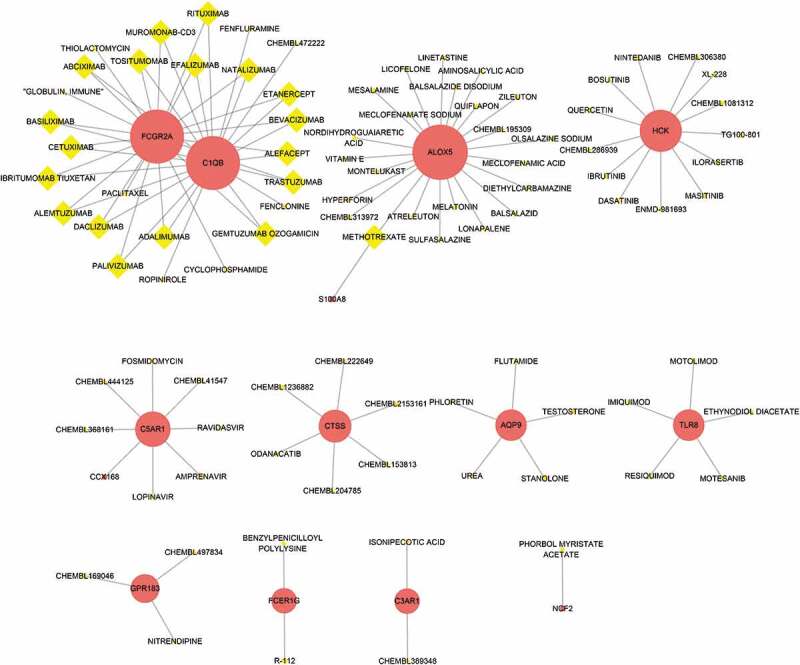
The drug-gene interaction network. The red circle nodes are the genes, and yellow diamond nodes are the drugs

DGIdb was applied to determine the potential drug or molecular compounds that could reverse the expression of down-regulated DEGs in the setting of bariatric surgery. As shown in the drug–gene interaction network (Figure 12), 18 drugs or molecular compounds included adalimumab, alefacept, and etanercept, which differentially regulated the expression of FCGR2A and C1QB. In addition, 23 drugs or molecular compounds, for instance, melatonin, nordihydroguaiaretic acid, and vitamin E, were found to interact with ALOX5. Further, five drugs or molecular compounds that included phloretin, regulated AQP9 and 13 drugs or molecular compounds that included ibrutinib and quercetin regulated HCK.

## 4. Discussion

Obesity shares a common characteristic with other prevalent, difficult-to-treat pathologies including the following: chronic, low-grade inflammation, which perpetuates the disease and is associated with multiple complications. The current interest in lipo-inflammation or chronic inflammation associated with obesity derives from an understanding of the alterations and remodelling that occurs in the adipose tissue, with the participation of multiple factors and elements throughout the process. Mounting evidence has demonstrated that bariatric surgery is an effective therapy for weight reduction, T2DM remission [37,38], cardiovascular risk factor improvement, other obesity-related co-morbidity remissions [39] and long-term survival improvement [17,40]. However, the molecular mechanisms on how bariatric surgery alleviates weight gain and precisely how these obesity complications and co-morbidities are resolved, at least to some clinical extent, are yet to be excavated.

In the present study, integrated bioinformatics methods assisted in an analysis of how critical genes change in their expression to uncover potential AT pathways before/after bariatric surgery based on two GEO datasets (i.e., GSE29409 and GSE59034), and we identified total overlap of 23 DEGs, including 22 down-regulated DEGs and 1 up-regulated DEG.

Further functional enrichment analyses were performed to clarify the role of down-regulated DEGs. BP analysis in GO annotation demonstrated that down-regulated DEGs after bariatric surgery were significantly enriched in the inflammatory response, which were mainly related to the immune response commonly associated with neutrophils, macrophages, and lymphocytes. Consistent with the previous evidence, obesity-induced inflammation is a low-grade chronic inflammation that is characterized by pro-inflammatory cells that include macrophages, neutrophils, Th1 CD4 + T cells, CD8 + T cells, natural killer T cells, and B-cells that infiltrate AT, thus playing a crucial role in insulin resistance and T2DM [41]. Moreover, B cells accumulating in adipose tissue in diet-induced obese (DIO) mice promote insulin resistance and do so by producing pathogenic IgG [42]. Besides, obesity-induced insulin resistance is driven by activation of endothelial FcγRIIB via hyposialylated IgG [43].

The previous results are in line with our MF analysis, which showed that IgG binding is the most significant GO term. KEGG enrichment analysis revealed that complement and coagulation cascades were a significant pathway. AT as an active metabolic and immune organ produces and regulates the plasma-borne complement level, thus promoting the generation of initiators of inflammation (e.g., complement component 3a and 5a). In proportion to the number of AT, anaphylatoxins like C3a and C5a trigger responses in the cyto/chemokine pathway, thus inducing inflammation and mediating metabolism associated with insulin resistance, diabetes, metabolic syndrome (MetS), and cardiovascular risk factors [44]. Besides, C3, C3a-desArg and C4 levels strongly correlate with lipid metabolism and the development of MetS and cardiovascular risk factors [45].

GSEA clarified a new perspective for this study. It demonstrated that such surgery-induced weight loss provokes a change from a state of obese pro-inflammation to anti-inflammation, which in turn improves metabolic functions. GSEA suggested that most of the genes in subjects before bariatric surgery were mainly enriched in the toll-like receptor signalling pathway, the NOD-like receptor signalling pathway, cytokine-cytokine receptor interactions, the chemokine signalling pathway, the B cell receptor signalling pathway, the Jak-STAT signalling pathway, which have collectively been confirmed as essential mechanisms in inflammation of AT, thus contributing to the pathogenesis of insulin resistance.

Also, two immune-related disease pathways that include autoimmune thyroid disease and type 1 diabetes mellitus were involved, which were confirmed by increasing studies in human, animal, and cell culture models, which showed that toll-like receptor signalling pathway activation acts as an essential trigger in promoting chronic inflammation and related metabolic disorders in AT, and does by activation of the myeloid diﬀerentiation primary response gene 88 signalling pathway that is triggered by saturated fatty acids [46].

The NOD-like receptor (NLR) protein family (NLRP3) in macrophage could be activated by lipid spill from necrotic adipocytes such as following the interplay of reactive oxygen species (ROS), saturated fatty acids, adenosine triphosphate, ceramides and dysfunction of the mitochondrial system, which is a predominant determinant in inflammation in AT and has critical functions in insulin resistance [47,48].

Abundant evidence has confirmed that chemokines are essential contributing elements in linking obesity and insulin resistance and metabolic comorbidities [49]. As previously described, B cells increase the inflammatory response in obesity and T2DM and do so by regulating the function of T cells and inflammatory cytokine profiles [50]. The JAK/STAT signalling pathway in AT plays a crucial role in regulating inflammation that is associated with metabolic abnormalities [51].

Besides, GSEA provides further evidence that the most significant-enriched gene sets that are positively correlated with subjects following bariatric were related to metabolism. Metabolites that directly sustain metabolic pathways; e.g., fatty acids, glucose and amino acids, also moderate immune cell function and metabolism [52]. Decreases in nutrient metabolites modulated by surgery lead to an anti-inflammation state and progression of metabolic functions by dampening pathological immune responses [53]. In addition, the reduction of adipocytes by bariatric surgery has been confirmed to dampen interactions with immune cells. Subsequently, reduction in the section of pro-inflammatory cytokines following activation of infiltrating immune cells in AT would similarly dampen activation of inflammation, and provoke instead a state of anti-inflammatory responsiveness and an improvement in insulin resistance, which in turn would improve the metabolism of glucose and generation of adipose. These mechanisms may account for how metabolic surgery prompts a significant shift from inflammation to metabolism.

Nine hub genes that include TYROBP, TLR8, FCER1 G, HCK, NCF2, CTSS, FCGR2A, MNDA, and C3AR1 were identified by constructing the PPI network and analysing it by MCODE and Cytohubba in Cytoscape. All of these hub genes were down-regulated after bariatric surgery.

TYROBP is involved in chronic inflammation via an association with the triggering receptors expressed by myeloid cells (TREM) in myeloid lineages [54]. For instance, the combined action of TREM 1 with TYROBP in macrophages or granulocytes increases the secretion of chemokines such as CCL2 and IL-8, and inflammatory cytokines like TNF-α [55]. TYROBP is involved in the inflammatory response and is negatively correlated with insulin sensitivity in AT [56]. The increased AT levels of TLR8 in obesity and T2DM were confirmed to be positively correlated with inflammatory markers like C-reactive protein and the expressions of inflammatory cytokines and chemokines. FCER1 G deficiency leads to reduced development of high-fat diet-induced obesity, which well explains AT inflammatory responses and IR at this time-point [57].

HCK plays a vital part in the progression of diabetes and does so by activating macrophages and subsequent secretion of TNF-α and inducible NO [58]. NCF2 expression increases with weight, participating in the development of ROS, which induces lipotoxicity [59]. An increasing number of vitro studies, as well as gene analysis, and clinical studies confirmed that Cathepsin S encoded by CTSS in AT is induced by inflammatory factors, which participates in the development of obesity and atherogenesis [60–62]. FCGR2A has been verified to be present on the adipocyte membrane in both omental and subcutaneous AT [63]. FCGR2A serves an essential role in atherosclerosis by combining with CRP, which leads to increased secretion of inflammatory cytokines, chemokines, and ROS [64]. MNDA is an inflammatory gene and is confirmed to be highly expressed in T1DM and hyperglycaemia [65,66]. C3AR1 is highly expressed in AT in the setting of obesity and atherosclerosis through the inflammatory response pathway [67].

Overall, these nine hub genes play a crucial role in the molecular level of activation of adipose inflammation, obesity, and the progression of obesity-associated co-morbidities, providing us some new potential therapeutic targets for obesity.

To further confirm the correlation between hub genes with obesity, the expression data of hub genes were acquired from Attie Lab and analysed. As anticipated, the expression of six genes that were collected from the above diabetes database and included, TYROBP, TLR8, FCER1 G, HCK, NCF2, CTSS, and C3AR1, were found to be higher in obesity as compared to the lean group. Subsequently, the methylation levels of hub genes were collected and analysed in DiseaseMeth. With the exception of HCK, which showed hypermethylation in T2DM, the remaining eight hub genes were confirmed to be hypomethylated and contributed to the explanation of unusual high expression levels of these hub DEGs. The expression of HCK could be subject to other reasons and thus warrant further research investigation.

In this study, the top three targeted DEGs in the miRNA-gene network were CYTIP, CECR1, and CXCL16. Studies in animals and humans showed that CXCL16 plays a key pro-inflammatory role in obesity and atherosclerosis, thereby increasing the progression of obesity [68,69]. Hsa-mir-26b-5p, which control the most DEGs, plays an essential role in adipogenesis [70]. Further research regarding the relation between CYTIP, CECR1 with obesity is clearly needed. The top five targeted DEGs in the TF-gene network were FCER1 G, TYROBP, C5AR1, CXCL16, and FCGR2A. As mentioned before, the complement and coagulation cascade pathways promote inflammation in adipocytes and obesity-associated co-morbidities, in which C5aR1 is a significant prevalent receptor [71]. The role of FCER1 G, TYROBP, CXCL16 and FCGR2A in obesity has been discussed previously.

To predict the potential effective therapy for obesity and obesity-related co-morbidities, we applied the DGIdb database to determine therapeutic agents that might reverse the abnormally high expression of obesity-related hub genes. Mounting epidemiological studies confirmed that TNF-α blockade, which includes the use of adalimumab (specific monoclonal antibodies) and etanercept (Fc fusion protein) improves MetS components (waist circumference, and serum lipid and glycaemic metabolism), and subsequently decreases the risk of T2DM and CVD in various inflammatory diseases such as psoriasis or rheumatoid arthritis (RA) [72–74]. Previously published studies showed that the efficacy of anti-TNF-α treatment in improving IR remains controversial [75]. Alefacept contributes to preserving β cell function in newly diagnosed T1DM [76]. Thiolactomycin is regarded as a promising template for new inhibitors of fatty acid synthase with an effect on weight loss. T2DM risk is associated with the defection of the melatonin signalling pathway [77]. Studies in animals and humans with metabolic disorders have demonstrated that melatonin supplementation improves IR and glycaemic control [78–80]. Nordihydroguaiaretic acid and phloretin have been confirmed to prevent high-fat-diet-induced metabolic dysfunction in mice. The efficacy of ibrutinib in lowering adipose inflammatory responses was shown in zebrafish studies [81]. Quercetin shows its anti-inflammatory, anti-obesity, and anti-diabetic effects in human and animal studies [82]. Vitamin E, especially tocotrienols (γ and δT3) [83], is a promising approach for anti-obesity and metabolic profile improvement through modulation of inflammation [84]. The roles of the drugs or molecular compounds above in obesity and their associated co-morbidities still need to be further explored as potential therapeutic targets.

There were some limitations in our study. First, the follow-up time for the two datasets was different (12 months for GSE29409 and 2 years for GSE59034). The CBS longitudinal cohort study [85] showed that substantial weight loss was sustainable for up to 2 years. Thus, our study aimed to identify whether mRNA significantly affects early changes in the expression of subcutaneous human AT of obese patients after bariatric surgery – at least in the short term (i.s., less than 3 years). However, such lack of consideration about follow-up time in detail may lead to some biological information being overlooked in our study. Second, though the method that high expression levels and hypomethylation of hub genes in obesity confirmed by the Attie Lab Diabetes database and DiseaseMeth 2.0, respectively, may not be optimal, should be sufficient to confirm the correlation between hub genes with obesity. However, our results cannot be validated due to the absence of experiments. Third, the data used in our study were accessed from a public database while the quality of the data cannot be appraised, and the method of Affymetrix gene expression arrays that was used in the data analyses for this study has not been used often. Last, the sample size of the involved data was relatively small, and the study failed to cover different racial/ethnic responses, and the impact of geographic disparities on the overall data analysis and conclusions, which can affect the analysis of gene expression before and after bariatric surgery in patients with obesity.

Despite these limitations, we conclude that bariatric surgery induces a significant shift from the state of obese pro-inflammation to a state of anti-inflammation, with improvement in adipocyte metabolic function, which represents key mechanisms whereby AT function improves after bariatric surgery. In the present study, we identified nine hub genes that were validated as highly expressed and hypomethylated in obesity, uncovered the possible pathways, analysed the target genes for miRNA/TF, and predicted potential therapeutic agents to explore the critical potential mechanisms that might plausibly be involved in such a metabolic surgery-induced switch of AT function through integrative analysis. Further studies are urgently warranted to verify and uncover further mechanisms. All of the current and future proposed output could empower the discovery of novel potential therapeutic targets to improve adipocyte inflammation – this paving the way for hopeful non-surgical treatment in the settings of obesity and obesity-related co-morbidities.

## Supplementary Material

Supplemental Material
