# Supplementary material for: Integrative analyses of biomarkers and pathways for adipose tissue after bariatric surgery
Source: Adipocyte. 2020 Jul 20;9(1):384–400. doi: 10.1080/21623945.2020.1795434 (PMC7469525; doi:10.1080/21623945.2020.1795434)
Supplement: Supplemental Material [file KADI_A_1795434_SM6492.docx]

**Table S1.** Gene ontology (GO) analysis of differentially expressed genes (DEGs) after bariatric surgery.

| Category | ID | GO Name | p value | p.adjust | q value | Gene | Count |
| --- | --- | --- | --- | --- | --- | --- | --- |
| BP | GO:0043312 | neutrophil degranulation | 2.05E-11 | 4.08E-09 | 2.38E-09 | FCGR2A/CTSS/ADA2/TYROBP/ALOX5/C5AR1/FCER1G/C3AR1/S100A8/MNDA | 10 |
| BP | GO:0002283 | neutrophil activation involved in immune response | 2.18E-11 | 4.08E-09 | 2.38E-09 | FCGR2A/CTSS/ADA2/TYROBP/ALOX5/C5AR1/FCER1G/C3AR1/S100A8/MNDA | 10 |
| BP | GO:0042119 | neutrophil activation | 2.66E-11 | 4.08E-09 | 2.38E-09 | FCGR2A/CTSS/ADA2/TYROBP/ALOX5/C5AR1/FCER1G/C3AR1/S100A8/MNDA | 10 |
| BP | GO:0002446 | neutrophil mediated immunity | 2.71E-11 | 4.08E-09 | 2.38E-09 | FCGR2A/CTSS/ADA2/TYROBP/ALOX5/C5AR1/FCER1G/C3AR1/S100A8/MNDA | 10 |
| BP | GO:0030595 | leukocyte chemotaxis | 1.89E-09 | 2.28E-07 | 1.33E-07 | JAML/C5AR1/GPR183/FCER1G/C3AR1/CXCL16/S100A8 | 7 |
| BP | GO:0060326 | cell chemotaxis | 1.59E-08 | 1.55E-06 | 9.02E-07 | JAML/C5AR1/GPR183/FCER1G/C3AR1/CXCL16/S100A8 | 7 |
| BP | GO:0050900 | leukocyte migration | 1.80E-08 | 1.55E-06 | 9.02E-07 | HCK/JAML/C5AR1/GPR183/FCER1G/C3AR1/CXCL16/S100A8 | 8 |
| BP | GO:0030593 | neutrophil chemotaxis | 6.39E-08 | 4.80E-06 | 2.80E-06 | JAML/C5AR1/FCER1G/C3AR1/S100A8 | 5 |
| BP | GO:1990266 | neutrophil migration | 1.07E-07 | 7.17E-06 | 4.18E-06 | JAML/C5AR1/FCER1G/C3AR1/S100A8 | 5 |
| BP | GO:0071621 | granulocyte chemotaxis | 1.52E-07 | 9.12E-06 | 5.32E-06 | JAML/C5AR1/FCER1G/C3AR1/S100A8 | 5 |
| BP | GO:0097530 | granulocyte migration | 2.63E-07 | 1.44E-05 | 8.39E-06 | JAML/C5AR1/FCER1G/C3AR1/S100A8 | 5 |
| BP | GO:0006968 | cellular defense response | 4.99E-07 | 2.50E-05 | 1.46E-05 | TYROBP/C5AR1/NCF2/MNDA | 4 |
| BP | GO:0097529 | myeloid leukocyte migration | 1.87E-06 | 8.62E-05 | 5.03E-05 | JAML/C5AR1/FCER1G/C3AR1/S100A8 | 5 |
| BP | GO:0002526 | acute inflammatory response | 2.49E-06 | 0.000107 | 6.23E-05 | C5AR1/FCER1G/C3AR1/S100A8/C1QB | 5 |
| BP | GO:0002429 | immune response-activating cell surface receptor signaling pathway | 3.61E-06 | 0.000144 | 8.43E-05 | HCK/FCGR2A/C5AR1/FCER1G/C3AR1/MNDA | 6 |
| BP | GO:0002758 | innate immune response-activating signal transduction | 4.04E-06 | 0.000148 | 8.65E-05 | HCK/CTSS/TLR8/FCER1G/S100A8 | 5 |
| BP | GO:0050727 | regulation of inflammatory response | 4.19E-06 | 0.000148 | 8.65E-05 | HCK/C5AR1/FCER1G/C3AR1/S100A8/C1QB | 6 |
| BP | GO:0002768 | immune response-regulating cell surface receptor signaling pathway | 5.46E-06 | 0.000182 | 0.000106 | HCK/FCGR2A/C5AR1/FCER1G/C3AR1/MNDA | 6 |
| BP | GO:0002218 | activation of innate immune response | 5.92E-06 | 0.000187 | 0.000109 | HCK/CTSS/TLR8/FCER1G/S100A8 | 5 |
| BP | GO:0045089 | positive regulation of innate immune response | 1.59E-05 | 0.000478 | 0.000279 | HCK/CTSS/TLR8/FCER1G/S100A8 | 5 |
| BP | GO:0002673 | regulation of acute inflammatory response | 1.77E-05 | 0.000508 | 0.000296 | C5AR1/FCER1G/C3AR1/C1QB | 4 |
| BP | GO:0006959 | humoral immune response | 2.86E-05 | 0.00078 | 0.000455 | C5AR1/GPR183/C3AR1/S100A8/C1QB | 5 |
| BP | GO:0045088 | regulation of innate immune response | 3.54E-05 | 0.000925 | 0.000539 | HCK/CTSS/TLR8/FCER1G/S100A8 | 5 |
| BP | GO:0031349 | positive regulation of defense response | 7.46E-05 | 0.001869 | 0.00109 | HCK/CTSS/TLR8/FCER1G/S100A8 | 5 |
| BP | GO:0002430 | complement receptor mediated signaling pathway | 8.61E-05 | 0.002069 | 0.001207 | C5AR1/C3AR1 | 2 |
| BP | GO:0010759 | positive regulation of macrophage chemotaxis | 0.000100335 | 0.002247 | 0.001311 | C5AR1/C3AR1 | 2 |
| BP | GO:0071674 | mononuclear cell migration | 0.000100964 | 0.002247 | 0.001311 | JAML/C5AR1/C3AR1 | 3 |
| BP | GO:0030316 | osteoclast differentiation | 0.000140518 | 0.003016 | 0.001759 | TYROBP/GPR183/FCER1G | 3 |
| BP | GO:1905523 | positive regulation of macrophage migration | 0.000149659 | 0.003102 | 0.001809 | C5AR1/C3AR1 | 2 |
| BP | GO:0007229 | integrin-mediated signaling pathway | 0.000158793 | 0.003181 | 0.001855 | HCK/TYROBP/FCER1G | 3 |
| BP | GO:0030449 | regulation of complement activation | 0.00019984 | 0.003858 | 0.00225 | C5AR1/C3AR1/C1QB | 3 |
| BP | GO:2000257 | regulation of protein activation cascade | 0.000205408 | 0.003858 | 0.00225 | C5AR1/C3AR1/C1QB | 3 |
| BP | GO:0032103 | positive regulation of response to external stimulus | 0.000257809 | 0.004695 | 0.002738 | C5AR1/FCER1G/C3AR1/S100A8 | 4 |
| BP | GO:0090023 | positive regulation of neutrophil chemotaxis | 0.000277329 | 0.004902 | 0.002859 | C5AR1/C3AR1 | 2 |
| BP | GO:0010758 | regulation of macrophage chemotaxis | 0.000302344 | 0.005067 | 0.002955 | C5AR1/C3AR1 | 2 |
| BP | GO:0016485 | protein processing | 0.000303502 | 0.005067 | 0.002955 | CTSS/C5AR1/C3AR1/C1QB | 4 |
| BP | GO:0002920 | regulation of humoral immune response | 0.000323456 | 0.005194 | 0.003029 | C5AR1/C3AR1/C1QB | 3 |
| BP | GO:0071624 | positive regulation of granulocyte chemotaxis | 0.000328422 | 0.005194 | 0.003029 | C5AR1/C3AR1 | 2 |
| BP | GO:1902624 | positive regulation of neutrophil migration | 0.00035556 | 0.005448 | 0.003177 | C5AR1/C3AR1 | 2 |
| BP | GO:0002224 | toll-like receptor signaling pathway | 0.000362593 | 0.005448 | 0.003177 | CTSS/TLR8/S100A8 | 3 |
| BP | GO:0010575 | positive regulation of vascular endothelial growth factor production | 0.000383755 | 0.005491 | 0.003203 | C5AR1/C3AR1 | 2 |
| BP | GO:0010818 | T cell chemotaxis | 0.000383755 | 0.005491 | 0.003203 | GPR183/CXCL16 | 2 |
| BP | GO:0090022 | regulation of neutrophil chemotaxis | 0.000413007 | 0.005755 | 0.003357 | C5AR1/C3AR1 | 2 |
| BP | GO:0038094 | Fc-gamma receptor signaling pathway | 0.000422336 | 0.005755 | 0.003357 | HCK/FCGR2A/FCER1G | 3 |
| BP | GO:0006909 | phagocytosis | 0.000440495 | 0.005755 | 0.003357 | HCK/FCGR2A/FCER1G/NCF2 | 4 |
| BP | GO:0002431 | Fc receptor mediated stimulatory signaling pathway | 0.000440497 | 0.005755 | 0.003357 | HCK/FCGR2A/FCER1G | 3 |
| BP | GO:1902622 | regulation of neutrophil migration | 0.000507077 | 0.006484 | 0.003782 | C5AR1/C3AR1 | 2 |
| BP | GO:0010574 | regulation of vascular endothelial growth factor production | 0.000540532 | 0.006768 | 0.003947 | C5AR1/C3AR1 | 2 |
| BP | GO:0045730 | respiratory burst | 0.000575032 | 0.006912 | 0.004031 | HCK/NCF2 | 2 |
| BP | GO:1905521 | regulation of macrophage migration | 0.000575032 | 0.006912 | 0.004031 | C5AR1/C3AR1 | 2 |
| BP | GO:0010573 | vascular endothelial growth factor production | 0.000610575 | 0.007195 | 0.004197 | C5AR1/C3AR1 | 2 |
| BP | GO:0051604 | protein maturation | 0.000642168 | 0.007339 | 0.00428 | CTSS/C5AR1/C3AR1/C1QB | 4 |
| BP | GO:0048246 | macrophage chemotaxis | 0.00064716 | 0.007339 | 0.00428 | C5AR1/C3AR1 | 2 |
| BP | GO:0006956 | complement activation | 0.000775503 | 0.008474 | 0.004942 | C5AR1/C3AR1/C1QB | 3 |
| BP | GO:0070613 | regulation of protein processing | 0.000775503 | 0.008474 | 0.004942 | C5AR1/C3AR1/C1QB | 3 |
| BP | GO:1903317 | regulation of protein maturation | 0.000802256 | 0.00861 | 0.005022 | C5AR1/C3AR1/C1QB | 3 |
| BP | GO:0002221 | pattern recognition receptor signaling pathway | 0.000900514 | 0.009495 | 0.005538 | CTSS/TLR8/S100A8 | 3 |
| BP | GO:0071622 | regulation of granulocyte chemotaxis | 0.000932239 | 0.009496 | 0.005539 | C5AR1/C3AR1 | 2 |
| BP | GO:0071675 | regulation of mononuclear cell migration | 0.000932239 | 0.009496 | 0.005539 | C5AR1/C3AR1 | 2 |
| BP | GO:0001819 | positive regulation of cytokine production | 0.000969043 | 0.009707 | 0.005661 | TLR8/C5AR1/FCER1G/C3AR1 | 4 |
| BP | GO:0038093 | Fc receptor signaling pathway | 0.001021822 | 0.010067 | 0.005872 | HCK/FCGR2A/FCER1G | 3 |
| BP | GO:1905517 | macrophage migration | 0.001117734 | 0.010669 | 0.006222 | C5AR1/C3AR1 | 2 |
| BP | GO:0002697 | regulation of immune effector process | 0.00112214 | 0.010669 | 0.006222 | C5AR1/FCER1G/C3AR1/C1QB | 4 |
| BP | GO:0072376 | protein activation cascade | 0.001136093 | 0.010669 | 0.006222 | C5AR1/C3AR1/C1QB | 3 |
| BP | GO:0002573 | myeloid leukocyte differentiation | 0.001187442 | 0.010979 | 0.006404 | TYROBP/GPR183/FCER1G | 3 |
| BP | GO:0050920 | regulation of chemotaxis | 0.001426899 | 0.012993 | 0.007578 | C5AR1/GPR183/C3AR1 | 3 |
| BP | GO:0016064 | immunoglobulin mediated immune response | 0.001466234 | 0.013135 | 0.007661 | TLR8/FCER1G/C1QB | 3 |
| BP | GO:0019724 | B cell mediated immunity | 0.001486155 | 0.013135 | 0.007661 | TLR8/FCER1G/C1QB | 3 |
| BP | GO:0030888 | regulation of B cell proliferation | 0.001832275 | 0.015731 | 0.009175 | GPR183/MNDA | 2 |
| BP | GO:0072678 | T cell migration | 0.001832275 | 0.015731 | 0.009175 | GPR183/CXCL16 | 2 |
| BP | GO:0002532 | production of molecular mediator involved in inflammatory response | 0.002086017 | 0.017412 | 0.010156 | ALOX5/FCER1G | 2 |
| BP | GO:0048247 | lymphocyte chemotaxis | 0.002086017 | 0.017412 | 0.010156 | GPR183/CXCL16 | 2 |
| BP | GO:0002292 | T cell differentiation involved in immune response | 0.002286648 | 0.018826 | 0.01098 | GPR183/FCER1G | 2 |
| BP | GO:0042116 | macrophage activation | 0.003338286 | 0.026751 | 0.015602 | TYROBP/TLR8 | 2 |
| BP | GO:0050764 | regulation of phagocytosis | 0.003338286 | 0.026751 | 0.015602 | HCK/FCER1G | 2 |
| BP | GO:0032755 | positive regulation of interleukin-6 production | 0.003420585 | 0.02705 | 0.015776 | TLR8/FCER1G | 2 |
| BP | GO:0002690 | positive regulation of leukocyte chemotaxis | 0.00367316 | 0.02867 | 0.016721 | C5AR1/C3AR1 | 2 |
| BP | GO:0001776 | leukocyte homeostasis | 0.004023097 | 0.030998 | 0.01808 | GPR183/FCER1G | 2 |
| BP | GO:0042100 | B cell proliferation | 0.004295362 | 0.032677 | 0.019059 | GPR183/MNDA | 2 |
| BP | GO:0042742 | defense response to bacterium | 0.004662578 | 0.035028 | 0.020429 | C5AR1/FCER1G/S100A8 | 3 |
| BP | GO:0007200 | phospholipase C-activating G protein-coupled receptor signaling pathway | 0.004864898 | 0.035688 | 0.020814 | C5AR1/C3AR1 | 2 |
| BP | GO:0032496 | response to lipopolysaccharide | 0.004869192 | 0.035688 | 0.020814 | HCK/C5AR1/S100A8 | 3 |
| BP | GO:0019886 | antigen processing and presentation of exogenous peptide antigen via MHC class II | 0.00496304 | 0.035937 | 0.02096 | CTSS/FCER1G | 2 |
| BP | GO:0002286 | T cell activation involved in immune response | 0.005262946 | 0.037212 | 0.021704 | GPR183/FCER1G | 2 |
| BP | GO:0002495 | antigen processing and presentation of peptide antigen via MHC class II | 0.005262946 | 0.037212 | 0.021704 | CTSS/FCER1G | 2 |
| BP | GO:0002504 | antigen processing and presentation of peptide or polysaccharide antigen via MHC class II | 0.005364736 | 0.037491 | 0.021866 | CTSS/FCER1G | 2 |
| BP | GO:0002237 | response to molecule of bacterial origin | 0.005432244 | 0.037526 | 0.021887 | HCK/C5AR1/S100A8 | 3 |
| BP | GO:0072676 | lymphocyte migration | 0.00567554 | 0.038761 | 0.022607 | GPR183/CXCL16 | 2 |
| BP | GO:0002449 | lymphocyte mediated immunity | 0.005751094 | 0.038836 | 0.022651 | TLR8/FCER1G/C1QB | 3 |
| BP | GO:0002688 | regulation of leukocyte chemotaxis | 0.006102559 | 0.040752 | 0.023768 | C5AR1/C3AR1 | 2 |
| BP | GO:0002460 | adaptive immune response based on somatic recombination of immune receptors built from immunoglobulin superfamily domains | 0.006225897 | 0.041118 | 0.023982 | TLR8/FCER1G/C1QB | 3 |
| BP | GO:0002687 | positive regulation of leukocyte migration | 0.007468977 | 0.048792 | 0.028457 | C5AR1/C3AR1 | 2 |
| BP | GO:0032675 | regulation of interleukin-6 production | 0.007709007 | 0.049818 | 0.029056 | TLR8/FCER1G | 2 |
| CC | GO:0030667 | secretory granule membrane | 1.57E-05 | 0.001068 | 0.000728 | FCGR2A/TYROBP/C5AR1/FCER1G/C3AR1 | 5 |
| CC | GO:0101002 | ficolin-1-rich granule | 4.86E-05 | 0.001654 | 0.001126 | CTSS/ALOX5/FCER1G/MNDA | 4 |
| CC | GO:0036019 | endolysosome | 0.000224384 | 0.004828 | 0.003288 | CTSS/TLR8 | 2 |
| CC | GO:1904813 | ficolin-1-rich granule lumen | 0.000345539 | 0.004828 | 0.003288 | CTSS/ALOX5/MNDA | 3 |
| CC | GO:0034774 | secretory granule lumen | 0.000404481 | 0.004828 | 0.003288 | ADA2/ALOX5/S100A8/MNDA | 4 |
| CC | GO:0060205 | cytoplasmic vesicle lumen | 0.000491504 | 0.004828 | 0.003288 | ADA2/ALOX5/S100A8/MNDA | 4 |
| CC | GO:0031983 | vesicle lumen | 0.000497007 | 0.004828 | 0.003288 | ADA2/ALOX5/S100A8/MNDA | 4 |
| CC | GO:0005766 | primary lysosome | 0.000663136 | 0.00501 | 0.003413 | ADA2/C3AR1/MNDA | 3 |
| CC | GO:0042582 | azurophil granule | 0.000663136 | 0.00501 | 0.003413 | ADA2/C3AR1/MNDA | 3 |
| CC | GO:0005775 | vacuolar lumen | 0.000881775 | 0.005996 | 0.004084 | CTSS/ADA2/MNDA | 3 |
| CC | GO:0031234 | extrinsic component of cytoplasmic side of plasma membrane | 0.00441366 | 0.026119 | 0.01779 | HCK/RGS1 | 2 |
| CC | GO:0035578 | azurophil granule lumen | 0.004609175 | 0.026119 | 0.01779 | ADA2/MNDA | 2 |
| CC | GO:0062023 | collagen-containing extracellular matrix | 0.005793178 | 0.030303 | 0.02064 | CTSS/S100A8/C1QB | 3 |
| MF | GO:0019864 | IgG binding | 7.38E-05 | 0.006202 | 0.003963 | FCGR2A/FCER1G | 2 |
| MF | GO:0019865 | immunoglobulin binding | 0.000336697 | 0.014141 | 0.009038 | FCGR2A/FCER1G | 2 |
| MF | GO:0043394 | proteoglycan binding | 0.000655835 | 0.018363 | 0.011736 | CTSS/ADA2 | 2 |

**Table S2.** Kyoto Encyclopedia of Genes and Genomes (KEGG) pathway analysis of differentially expressed genes (DEGs) after bariatric surgery.

| Pathway ID | Pathway Name | p value | p.adjust | q value | Gene | Count |
| --- | --- | --- | --- | --- | --- | --- |
| hsa05150 | Staphylococcus aureus infection | 2.47E-05 | 0.000888 | 0.000623 | FCGR2A/C5AR1/C3AR1/C1QB | 4 |
| hsa04610 | Complement and coagulation cascades | 0.00049 | 0.008818 | 0.006188 | C5AR1/C3AR1/C1QB | 3 |
| hsa04380 | Osteoclast differentiation | 0.001612 | 0.019349 | 0.013578 | FCGR2A/TYROBP/NCF2 | 3 |
| hsa04145 | Phagosome | 0.002637 | 0.023735 | 0.016656 | FCGR2A/CTSS/NCF2 | 3 |
| hsa04664 | Fc epsilon RI signaling pathway | 0.007052 | 0.04231 | 0.029691 | ALOX5/FCER1G | 2 |
| hsa05140 | Leishmaniasis | 0.008969 | 0.046127 | 0.03237 | FCGR2A/NCF2 | 2 |
